# Modulating Inhibitory Control Processes Using Individualized High Definition Theta Transcranial Alternating Current Stimulation (HD θ-tACS) of the Anterior Cingulate and Medial Prefrontal Cortex

**DOI:** 10.3389/fnsys.2021.611507

**Published:** 2021-03-30

**Authors:** Monika Klírová, Veronika Voráčková, Jiří Horáček, Pavel Mohr, Juraj Jonáš, Daniela Urbaczka Dudysová, Lenka Kostýlková, Dan Fayette, Lucie Krejčová, Silvie Baumann, Olga Laskov, Tomáš Novák

**Affiliations:** ^1^National Institute of Mental Health, Prague, Czechia; ^2^Department of Psychiatry, Third Faculty of Medicine, Charles University, Prague, Czechia

**Keywords:** transcranial alternating current stimulation, tACS, ACC, theta frequency, inhibitory control, high definition

## Abstract

Increased frontal midline theta activity generated by the anterior cingulate cortex (ACC) is induced by conflict processing in the medial frontal cortex (MFC). There is evidence that theta band transcranial alternating current stimulation (θ-tACS) modulates ACC function and alters inhibitory control performance during neuromodulation. Multi-electric (256 electrodes) high definition θ-tACS (HD θ-tACS) using computational modeling based on individual MRI allows precise neuromodulation targeting of the ACC via the medial prefrontal cortex (mPFC), and optimizes the required current density with a minimum impact on the rest of the brain. We therefore tested whether the individualized electrode montage of HD θ-tACS with the current flow targeted to the mPFC-ACC compared with a fixed montage (non-individualized) induces a higher post-modulatory effect on inhibitory control. Twenty healthy subjects were randomly assigned to a sequence of three HD θ-tACS conditions (individualized mPFC-ACC targeting; non-individualized MFC targeting; and a sham) in a double-blind cross-over study. Changes in the Visual Simon Task, Stop Signal Task, CPT III, and Stroop test were assessed before and after each session. Compared with non-individualized θ-tACS, the individualized HD θ-tACS significantly increased the number of interference words and the interference score in the Stroop test. The changes in the non-verbal cognitive tests did not induce a parallel effect. This is the first study to examine the influence of individualized HD θ-tACS targeted to the ACC on inhibitory control performance. The proposed algorithm represents a well-tolerated method that helps to improve the specificity of neuromodulation targeting of the ACC.

## Introduction

The anterior cingulate cortex (ACC) plays an important role in the processing of cognition and emotions (Gasquoine, 2013; Onoda et al., 2017). While cognitive processes are attributed to the dorsal ACC (dACC), emotional processes are assigned to the rostral division of the ACC (Bush et al., 2000). According to recent comprehensive theory, the ACC is activated in response to a conflict between incompatible streams of information processing; it represents an essential structure for the inhibitory control process (van Veen and Carter, 2002). ACC disruptions in various neuropsychiatric disorders, such as schizophrenia and obsessive-compulsive disorder (OCD), have been documented by functional magnetic resonance imaging (fMRI) studies (Carter et al., 2001; Ursu et al., 2003; Fitzgerald et al., 2005). In schizophrenia, the reduction of ACC activity results in decreased activity in the processing of conflicting information and inhibition ability (Carter et al., 2001), while in OCD, higher ACC activity increases conflict-processing information and inhibitory activity (van Veen and Carter, 2002; Ursu et al., 2003; Fitzgerald et al., 2005; Gasquoine, 2013).

The medial prefrontal cortex (mPFC), also covering the dACC, generates theta oscillatory activity, so-called Frontal Midline Theta (FMT). FMT enhancement over the medial frontal cortex (MFC), recorded during conflict monitoring, error processing, and top-down behavior adjustments (Cohen et al., 2008; Cavanagh and Shackman, 2015; Van Noordt et al., 2016) has also been documented as having behaviorally relevant event-related potentials (ERPs) during resolution of conflict-related tasks (Nigbur et al., 2012; Cohen and Donner, 2013) associated with the procession of inhibitory control. The latter can be evaluated by verbal (Stroop Test) or non-verbal (Stop Signal, Visual Simon, or Flanker Task) tests (Kopp et al., 1996a,b; Gratton et al., 1988; Bush et al., 2000; Taylor et al., 2007; Cohen and Donner, 2013; Spielberg et al., 2015), measuring different parameters, such as commission of mistakes or those related to conflict and error.

Transcranial Alternating Current Stimulation (tACS) enables modulation of endogenous oscillations at the pacing rate (Paulus, 2011; Ali et al., 2013; Antal and Paulus, 2013; Herrmann et al., 2013; Antal and Herrmann, 2016), inducing frequency-specific activity changes (Vossen et al., 2015; Witkowski et al., 2016) and the subsequent induction of synaptic changes via neuronal plasticity, providing a post-modulation effect on brain oscillation activity (Antal et al., 2008; Zaehle et al., 2010; Vossen et al., 2015; Berger et al., 2018; Moliadze et al., 2019). The effect of various tACS frequencies on cognition was tested (Sela et al., 2012; Hoy et al., 2015; van Driel et al., 2015; Vosskuhl et al., 2015; Wischnewski et al., 2016; Fusco et al., 2018; Pahor and Jaušovec, 2018; Lehr et al., 2019; Moliadze et al., 2019). Specific cognitive abilities were significantly modified mostly by the theta frequency tACS (θ-tACS; Sela et al., 2012; van Driel et al., 2015; Vosskuhl et al., 2015; Wischnewski et al., 2016; Fusco et al., 2018, 2020; Pahor and Jaušovec, 2018; Lang et al., 2019; Lehr et al., 2019). Neuroimaging (MEG and fMRI) studies have also shown that FMT performance is influenced by θ-tACS, which modulates dACC network functions (Chander et al., 2016; Onoda et al., 2017). However, previous studies that documented the behavioral and electrophysiological effects of θ-tACS have mostly targeted various non-specific anatomic frontal cortex regions (van Driel et al., 2015; Vosskuhl et al., 2015; Chander et al., 2016; Onoda et al., 2017; Fusco et al., 2018; Pahor and Jaušovec, 2018), including the MFC (van Driel et al., 2015; Vosskuhl et al., 2015; Fusco et al., 2018).

So far, only a few studies have tested the impact of θ-tACS on inhibitory control performance (van Driel et al., 2015; Fusco et al., 2018, 2020; Lehr et al., 2019). For example, Lehr et al.’s (2019) study targeted the dorsolateral prefrontal cortex (DLPFC), which together with the ACC belongs to the inhibitory control circuit. Other studies (van Driel et al., 2015; Fusco et al., 2018, 2020) targeted the ACC indirectly via the MFC. With the exception of van Driel et al.’s (2015) study, these experiments monitored the effect of θ-tACS on inhibitory control performance only during the application of θ-tACS; the post-modulatory effect was not tested (Fusco et al., 2018, 2020; Lehr et al., 2019). The studies have yielded inconsistent results, ranging from enhancement (Fusco et al., 2018, 2020) to deterioration of inhibitory control performance (van Driel et al., 2015; Lehr et al., 2019).

It has been suggested that tACS may be used as a tool to investigate diseases with altered EEG activity (Antal et al., 2008; Ahn et al., 2019; Del Felice et al., 2019). Therefore, the effect of θ-tACS of the ACC on inhibitory control could be examined in neuropsychiatric disorders associated with disrupted dACC activity. We assume that θ-tACS targeted directly at the source of FMT via the mPFC-ACC may modify inhibitory control processes with a sustained effect.

However, standard transcranial Electrical Stimulation (tES; Nikolin et al., 2015) technologies (in terms of electrode assembly, number, shape, and size) are insufficient for more specific targeted tES (Lefaucheur et al., 2017); precise targeting of the deeper brain structures cannot therefore be documented. In addition, standard tES directly affects not only the selected area of interest but also the surrounding and interconnected structures.

High definition (HD) tES provides a solution to these methodological limitations. It is guided by structural neuroimaging, which allows a computer model of the electric field intensity distributions to be created. This model is essential for precise neuromodulation targeting with respect to the different conductivity of various brain tissues (Nikolin et al., 2015; Alam et al., 2016). Geodesic Transcranial Electrical Neuromodulation (GTEN) system with 256 electrodes enables Multielectrode Transcranial Electrical Stimulation (MTES), which optimizes the required current density in the targeted brain area. MTES analyses (Dmochowski et al., 2011; Alam et al., 2016; Fernández-Corazza et al., 2016) have shown that the use of a higher electrode density improves the focus, directionality, and stimulation intensity parameters by penetrating the deeper brain structures. MTES enables targeted neuromodulation of selected areas with greater specificity than the standard technologies; therefore, it is possible to modulate the ACC activity via mPFC-ACC stimulation more selectively.

Our study goal was to investigate the post-modulatory effects of θ-tACS in inhibitory control processing. For this purpose, we applied different θ-tACS neuromodulation protocols targeted at the MFC in healthy subjects, and evaluated θ-tACS-induced post-modulatory changes in tasks specific to inhibitory control. We assumed that by strengthening an oscillating current at the theta frequency, θ-tACS would induce a persistent improvement of the inhibitory control performance in the post-modulation assessment. We hypothesized that the individualized HD θ-tACS, targeted according to the computational modeling for optimal electrode arrangement, would induce a post-modulatory effect of θ-tACS on inhibitory control. This effect would be significantly stronger than the effect of a non-individualized θ-tACS targeting the ACC indirectly via the MFC. Moreover, we also compared the effect of individualized HD θ-tACS with non-individualized θ-tACS and with a sham condition.

## Materials and Methods

### Subjects

Twenty healthy volunteers (mean age 34.4 ± 7.2 years, 10 females) were included in the study. Exclusion criteria were a current diagnosis or history of a psychiatric disorder; substance use disorder (with the exception of nicotine); regular use of any medication that might affect cognitive functions (e.g., antihistamines and benzodiazepines); a history of serious head injury or a neurological disorder; a medical condition that could interfere with the tES administration (Poreisz et al., 2007); pregnancy; breastfeeding; or a sensory or motor impairment. All participants signed an informed consent form in accordance with the latest version of the Declaration of Helsinki; the study protocol was approved by the Independent Ethics Committee of the National Institute of Mental Health, Klecany.

### Study Protocol

**FIGURE 1:**
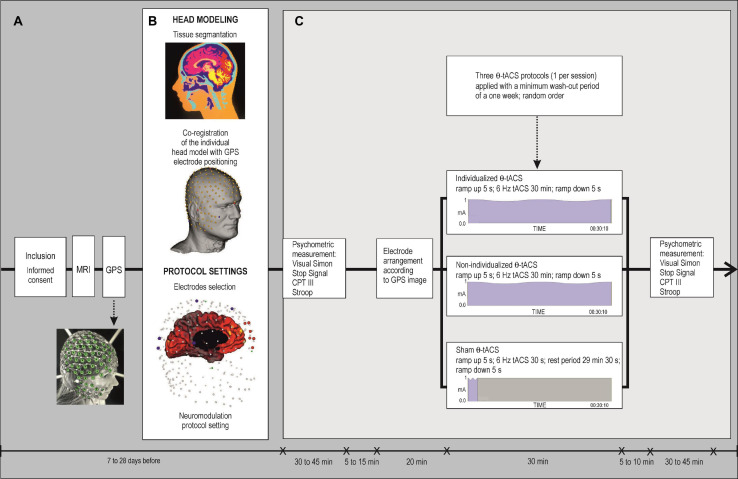
Flow chart of the protocol. **(A)** Schematic overview of the study. **(B)** Illustration of head modeling with tissue segmentation, co-registration of an individual head model with GPS electrode positioning, and setting of a protocol to select the appropriate electrodes according to the individual cortical current density, followed by neuromodulation protocol settings. **(C)** Experimental procedure. A double-blind, cross-over study. Three neuromodulation sessions were carried out in random order with a minimum 1 week washout period. The psychometric measurement was tested immediately before and after the end of each neuromodulation session. Types of HD θ-tACS protocols: an individualized HD θ-tACS was targeted with the highest specificity to the mPFC-ACC; a non-individualized θ-tACS was targeted over the MFC with fixed electrode positions in the FCz and Pz areas; a sham tACS was administered with identical electrode montage (as used in the non-individualized protocol). Active θ-tACS sessions comprised a 5 s ramp up, 30 min of 6 Hz θ-tACS, and a 5 s ramp down. Sham condition comprised a 5 s ramp up and 30 s of 6 Hz θ-tACS followed by a 29 min 30 s rest period and a 5 s ramp down. Psychometric measurement was assessed by the Simon Task, Stop Signal Task, Conners’ Continuous Performance Test 3rd edition (CPT III), and the Stroop Test.

A double-blind, sham-controlled, three-condition, three-period cross-over study was carried out in random order, with a 1 week minimum wash-out period to avoid carryover effects. Following Williams’ design (Williams, 1949) for the 3 × 3 cross-over trial, participants were randomly assigned to one of six different sequences of three θ-tACS conditions: (1) individualized targeting of the mPFC-ACC according to the individual head model; (2) non-individualized electrodes placed in the FCz and Pz areas according to the skull’s anatomy (Vosskuhl et al., 2015; Fusco et al., 2018); and (3) the sham (Figure 1A). To assure a balanced number of subjects in each sequence, we used non-stratified blocked randomization with a block size of six (computer generated^1^). Every experimental session for each subject was carried out at the same time of day, on the same weekday. Participants were asked to abstain from caffeine and nicotine for at least 2 h prior to the cognitive assessment and the tACS session and to refrain from alcohol or any medication for 24 h prior to the tACS administration.

The study participants and the researchers evaluating the psychometric measurements remained blind to the stimulation conditions and parameters. Neuropsychological assessment of inhibitory control was performed before and after each tACS application (Figure 1C). To test for the integrity of the blinding procedure, all participants were asked the following question at the end of the study: “During which stimulation session was a placebo applied? Was it during the first, second, or third session?” There were three possible answers: a placebo was applied during the first (011), the second (101), or the third (110).

### Magnetic Resonance Imaging (MRI) and the Geodesic Photogrammetry System (GPS)

Individual magnetic resonance images obtained with a Siemens Magnetom Prisma 3T system (Siemens, Erlangen, Germany) were used to create an individual head model for each subject. The subjects were scanned using a T1 Sag MPR 1 mm ISO sequence (repetition time TR of 2,300 ms; echo time TE of 1.69 ms; field of view 288 mm; slice slab 256 mm; voxel size of 1 × 1 × 1 mm and total acquisition time 5 min 12 s).

Subsequently, to obtain accurate positions for the individual electrodes on the scalp, the head of the subject was targeted by a sensor-registration system determining the 3-dimensional position of each of 256 sensors (neuromodulation electrodes). Electrode sensor positions of the 256-channel HydroCel Geodesic Sensor Net (HCGSN) 100 (EGI, Eugene, OR, United States) were digitalized with a GPS (EGI, Eugene, OR, United States) (Luu et al., 2016).

### Head Modeling

Figure 1B schematically illustrates the head modeling with tissue segmentation, co-registration of an individual head model with GPS electrode positioning, and the settings of a protocol to select the appropriate electrodes according to the individual cortical current density followed by the neuromodulation protocol settings.

The individual head models were processed by the Modal Image Pipeline, ver. 1.10 (EGI, Eugene, OR, United States). To calculate the accurate head model, the initial segmentation of the white matter, gray matter, and background ratio was processed. Particular attention was given to the manual correction of gray matter smoothing and cortical surface extraction, to avoid digital errors when calculating normal the cortical conductivity vectors (Fernández-Corazza et al., 2016). The MRI images were subsequently segmented into tissue types (scalp, skull, eyeballs, cerebrospinal fluid, gray matter, white matter, and air) and proportionalized to the right and left hemispheres. Following the scalp reconstruction necessary for co-registration with the GPS sensors, the landmarks corresponding to the exact positions of selected electrodes from the individual GPS images were marked on the head model. In the next step, a 3D cortical surface with a 4800 dipoles patch was created to describe the current flow from the scalp to the cortex (Fernández-Corazza et al., 2016; Luu et al., 2016). The head model of the cortical surface was subsequently co-registered in JavaScript with a GPS file containing the photographic images of the individual positions of each of the 256 sensors placed on the subject’s head while images were taken by the GPS camera.

The final head model with a neuromodulatory electrode selection and stimulation protocol setting was designed in the Reciprocity Visualization Environment, ver. 1.1 (EGI, Eugene, OR, United States), which allows the optimal electrode arrangement to be selected and the appropriate amount of current delivered by the current injection through each electrode (Luu et al., 2016) to be determined to achieve the maximum current density at a given target of the cortical surface model.

### Neuromodulation Protocols

**FIGURE 2:**
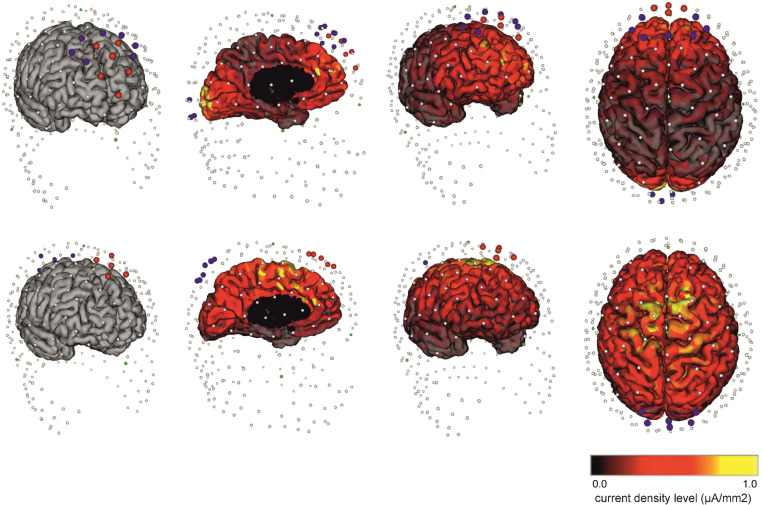
The electrode placement used in the individualized and non-individualized HD θ-tACS protocols. **(Top)** The individualized placement of electrodes for HD θ-tACS of the ACC targeted through the mPFC-ACC was selected on the basis of a reciprocity-optimized selection of current injection electrodes. Six anode-cathodes (red electrodes) and 10 cathode-anodes (blue electrodes) are shown. Note that to select the optimal electrode arrangement and current injection electrode, the individualized flux pattern with the highest specificity of current flow for a given target region (mPFC-ACC) on the cortical surface model was used. This is an example montage. **(Bottom)** Non-individualized placement of electrodes in the FCz and Pz areas (Vosskuhl et al., 2015; Fusco et al., 2018) adapted for the HCGSN 100 using the 10-channel mount with five anode-cathodes (red electrodes) in the FCz area and five cathode-anodes (blue electrodes) in the Pz area for a MFC and medial parietal cortex electrode layout. **(Left)** anterior view without marking the current density; **(middle left)** sagittal view; **(middle right)** anterior view; and **(right)** view from above, with cortex coloring representing the calculated current density on the cortical surface from 0.0 μA/mm^2^ (black) to 1.0 μA/mm^2^ (yellow).

An optimal electrode arrangement based on computational modeling from individual MRI was used for the individualized protocol with the current flow targeted with the highest specificity at the mPFC-ACC, and to achieve maximum current density in the ACC. A mounting (up to 16 channels) with a maximum of six cathode-anodes and 10 anode-cathodes was employed, because the anatomical variations of the subjects would have a potentially significant impact on the field strength in a given area. The specification of the electrode layouts, including the number of electrodes, their position, and current intensity in each electrode for individualized tACS for each subject is shown in Table 1. The arrangement of the neuromodulatory electrodes for the individual head models was computed with respect to individual cortical geometry, the head shapes of the subjects, and the corresponding current densities of the cortical surface (Figure 2).

**TABLE 1:** The specification of electrode layouts (256-channel HydroCel Geodesic Sensor Net), including the number of electrodes, their position, and current intensity in each electrode used for individualized HD θ-tACS in subjects.

**No.**	**Sex**	**No. A/C**	**No. C/A**	**No. A/C + C/A**	**A/C Layout (200 μA)**	**A/C Layout (100 μA)**	**C/A Layout (200 μA)**	**C/A Layout (100 μA)**	**C/A Layout (50 μA)**
1	2	6	10	16	20; 21 (Fz); 26; 27	14; 22	124; 125; 137	15	5; 6; 13; 23;28; 29
2	2	6	10	16	20; 21 (Fz); 26; 27	14; 22	124; 125; 137	15	5; 6; 13; 23;28; 29
3	2	6	10	16	20; 21 (Fz); 26; 27	14; 22	124; 125; 137	15	5; 6; 13; 23;28; 29
4	1	6	10	16	20; 21 (Fz); 26; 27	14; 22	124; 125; 137	15	5; 6; 13; 23;28; 29
5	2	6	10	16	20; 21 (Fz); 26; 27	14; 22	124; 125; 137	15	5; 6; 13; 23;28; 29
6	2	6	10	16	20; 21 (Fz); 26; 27	14; 22	124; 125; 137	15	5; 6; 13; 23;28; 29
7	1	6	10	16	20; 21 (Fz); 26; 27	14; 22	124; 125; 137	15	5; 6; 13; 23;28; 29
8	2	5	8	13	21; 26; 27; 28; 33		117; 125; 126 (Oz)	16; 70; 182	22; 32;
9	1	6	10	16	20; 21 (Fz); 26; 27	14; 22	124; 125; 137	15	5; 6; 13; 23;28; 29
10	1	5	6	11	20; 26; 27; 31 (NAS)	25; 32		15; 64; 81; 180	126 (Oz); 138; 139
11	2	5	10	15	20; 21 (Fz); 26; 27	14; 22	124; 125; 137	15	5; 6; 13; 23;28; 29
12	1	6	10	16	20; 21 (Fz); 26; 27	14; 22	124; 125; 137	15	5; 6; 13; 23;28; 29
13	2	6	10	16	20; 21 (Fz); 26; 27	14; 22	124; 125; 137	15	5; 6; 13; 23;28; 29
14	1	6	10	16	20; 21 (Fz); 26; 27	14; 22	124; 125; 137	15	5; 6; 13; 23;28; 29
15	2	5	7	12	20; 21 (Fz); 26; 27	14; 22	124; 125; 137	15	5; 6; 13; 23;28; 29
16	1	6	6	12	20; 26; 27; 31 (NAS)	25; 32		15; 64; 81; 180	126 (Oz); 138; 139
17	1	5	5	10	19; 20; 21; 25; 26		70; 125; 126 (Oz); 138; 179		
18	1	6	10	16	20; 21 (Fz); 26; 27	25; 32	137; 138; 148; 149		13; 14; 22; 28
19	2	5	6	11	20; 26; 27; 31 (NAS)	25; 32		15; 64; 81; 180	126 (Oz); 138; 139
20	1	5	7	12	20; 26; 27; 31 (NAS)	25; 32		15; 64; 81; 180	126 (Oz); 138; 139

**Sex: 1 (male); 2 (female). Neuromodulatory electrode specification: A/C, cathode-anodes; C/A, anode-cathodes; μA defines the electric current used for each electrode. A total current 1 mA for each group of electrodes (A/C and C/A) was used for each subject. Fz, Midline Frontal; Oz, Midline Occipital; NAS, Nasion.**

The non-individualized focusing of the electrode assembly (according to the skull anatomy) used MFC and medial parietal cortex electrode layouts (Vosskuhl et al., 2015; Fusco et al., 2018), specifically in the FCz and Pz areas (according to the international 10/20 EEG electrode system for “standard” anatomically guided positioning), and adapted for HCGSN 100 using the 10-channel mount with five anode-cathodes in the FCz area and five cathode-anodes in the Pz area (Figure 2).

The sham tACS was administered with an identical electrode montage, as used in the non-individualized protocol.

### tACS Administration

Electrode positioning of the 256-channel HCGSN 100 was adjusted under the control of the GPS subordinate images prior to each tACS application. An Elefix conductive paste (Nihon Kohden, Tokyo, Japan) was used as the conductive material between the scalp and selected neuromodulatory electrodes. The impedance of the electrodes was monitored immediately before tACS application; it was below 10 KΩ.

Subsequently, a pulsed alternating current was applied with Net Station Acquisition software, ver. 5.4.2 (EGI, Eugene, OR, United States), using a CE-certified Geodesic Transcranial Electrical Neuromodulation (GTEN) 100 system (EGI, Eugene, OR, United States) with the 256-channel HCGSN 100, which is an evenly spaced network of Ag-AgCl electrodes (Luu et al., 2016). The maximum current at any given electrode (1 cm^2^) was 200 μA, with a total current of tACS 1 mA for each active session, under monitoring by a GTEN 100 Sentinel Circuit^®^.

The parameter settings for each active θ-tACS session were: a 5 s ramp up, 30 min of θ-tACS with 6 Hz, and a 5 s ramp down. For the sham condition, the application of θ-tACS after a 5 s ramp up was shortened to 30 s and was followed by a rest period of 29 min 30 s, terminated by a 5 s ramp down. A sham condition applied for a duration of 30 s has been previously described as a reliable blinding method for tACS (Zaghi et al., 2010; Pahor and Jaušovec, 2018). This reproduces somatic sensations similar to active stimulation. Tingling or heating up of the scalp occurs mostly at the beginning (ramp up and initial adaptation to θ-tACS) of the neuromodulation and at the end of the protocol (ramp down). During the θ-tACS application, the participants were in a resting condition (Figure 1C).

### Psychometric Measurement

**FIGURE 3:**
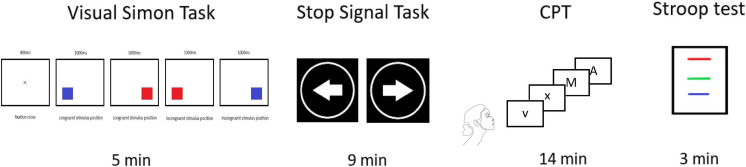
Flow chart of the psychometric measurement. Flow chart of the psychometric measurement. Illustration of the individual assessments performed for the following periods: Visual Simon Task = 5 min, Stop Signal Task = 9 min, Conners’ Continuous Performance Test 3rd edition (CPT III) = 14 min, Stroop test = 3 min.

Psychometric measurements were performed immediately before and after each neuromodulation session by a rater blind to the treatment condition. The neuropsychological battery consisted of the following non-verbal tests: Visual Simon Task (Simon Task), Stop Signal Task, Conners’ Continuous Performance Test 3rd edition (CPT III), and verbal tests: Stroop Color and Word Test (SCWT or Stroop; Figure 3). The tests focused primarily on specific parameters of the cognitive domains that are related to cognitive control and that have shown minimum practice effect. The Simon Task and the Stop Signal Task were administered using the program Inquisit 3 (Inquisit, 2007).

The Simon Task is used to measure the stimulus-response compatibility and the difference in reaction time (RT) between trials in which the prepotent association is congruent to the stimulus and the trials in which it is incongruent (Bialystok et al., 2004). Each trial of the task begins with a central fixation cross on the screen (800 ms), followed by a blank interval (250 ms). Then, a red or blue square appears on either the left or the right side of the screen and remains there if there is no response (1,000 ms). Participants are required to press the A key (left) when a blue square is presented or the L key (right) when a red square is presented. Response timing starts with the onset of the stimulus, and the participant’s response terminates the stimulus. The task takes approximately 2 min to complete, and involves 5 min of instruction. Participants have to complete all eight practice trials without a mistake before the experimental trials begin. If participants fail to press the key within the available time, they receive additional practice trials until all eight attempts are completed correctly. The following experiment (28 trials) consists of 14 congruent trials (presenting the square on the same side as the related response key) and 14 incongruent trials (presenting the square on the opposite side), which are presented in random order (Inquisit, 2007).

The Stop Signal Task was selected to assess the ability to stop a planned or ongoing action (van Gaal et al., 2009). It is a go/nogo reaction time task that provides a means of estimating the time it takes to stop executing a response that might already be underway but needs to be halted. This is called the stop signal reaction time. At the beginning of the task, a fixation circle is displayed in which an arrow points to the left or to the right. Participants are required to press the “←” key or “→” key, depending on the direction of the arrow, unless a beep sounds to stop the response before execution. Depending on performance, the delay between the appearance of the arrow and the beep sound is adjusted up or down. Previous successful trials make the delay longer in the new trial and previous unsuccessful trials make it shorter. Participants’ responses are available until the end of the trial. Each task trial has a duration of 2,000 ms. The initial Stop Signal Delay (SSD) is set to 250 ms, and between 50 to 1,150 ms with adjustment steps of 50 ms thereafter. The task takes approximately 9 min to complete. Arrows with different directions (half to the right; half to the left) are presented in random order. The practice block includes 32 trials (8 signal trials and 24 no signal trials). The following three experimental blocks consist of 72 trials each (18 signal trials and 54 no signal trials; Inquisit, 2007).

CPT III, which is designed primarily to assess attention-related changes (Conners, 2014), was used to measure expected changes in commissions (incorrect response to non-targeted), perseverations (number of perseverative responses represented by responses with a reaction time of less than 100 ms); hit reaction time (average speed of correct responses), hit reaction time (HRT) block change (the slope of change in HRT across the six blocks of the administration), and detectability (the ability of a respondent to discriminate non-target from other targets) scores. During the task, participants are required to press the spacebar when any letter appears (with the exception of “X”). The interstimulus interval between each letter is 1, 2, or 4 s. The test takes 14 min to complete and consists of 18 blocks with 20 trials (360 in total).

The classic card version of the Stroop assesses the test parameters associated with inhibitory control, specifically the difficulty in warding off distractors (interference words) in the color-word (CW) condition (Lezak et al., 2004), where successful performance requires the ability to inhibit pre-potent verbal responses and conflict monitoring (Cipolotti et al., 2016), and the Stroop Interference Score (IG), a value obtained by subtracting the “predicted CW score” from the actual CW score; this reflects an ability to inhibit interference. The test consists of three subtests/charts: a word (W) chart, a color (C) chart, and a chart with colored words (CW). Each chart contains 100 items arranged in five columns of 20 items, printed on a white background. The individual charts are administered in the order W, C, and CW. The W chart consists of the words “red,” “green,” and “blue,” printed in black. The words are arranged in random order so that two identical words do not follow each other within the column. C chart consists of the color items “XXX,” which are printed in a corresponding color: red, green, or blue. The color of two consecutive items is always different. The CW chart contains colored words in the same order as the W cart, but no item is printed in a color corresponding to the meaning of the word. The time limit on each chart is 45 s. In total, even with the instruction, the test takes approximately 3 min.

In addition, a qualitative questionnaire of adverse effects, including bodily sensations and subjective mood changes induced by θ-tACS was administered during and after each neuromodulation session.

### Statistical Analyses

All analyses were conducted using the software package Stata, ver. 15 (StataCorp, 2017; College Station, TX: StataCorp LLC). The tests were two-sided and *p* < 0.05 was regarded as being statistically significant. Differences in the effect of tACS conditions (individualized [A_1_], non-individualized [A_2_], and sham [S]) on cognitive performance (Visual Simon Task, Stop Signal Task, CPT III, and Stroop Test) were analyzed in a linear mixed-effect model fitted by a restricted maximum likelihood procedure and with a Kenward-Roger degrees of freedom approximation. In the model, sequence (six sequences: A_1_A_2_S, A_2_SA_1_, SA_1_A_2_, A_1_SA_2_, A_2_A_1_S, and SA_2_A_1_), period (three periods: sessions 1, 2, and 3), first-order carryover, and treatment (three tACS conditions: A_1_, A_2_, and S) were entered as fixed effects, baseline performance as a continuous covariate, subject within sequence as a random effect, and covariance structure as unstructured. Additionally, *post hoc* differences in least-square means (and 95% confidence intervals) between treatment conditions were obtained. Pairwise comparisons with Sidak’s multiple testing adjustment were applied.

To assess blinding integrity we compared agreement/disagreement using Cohen’s kappa with an expected chance agreement of 34.3%. To assess the side effects of θ-tACS, including bodily sensations or mood changes, the numbers of participants who experienced them were compared across treatment conditions using a Cochrane *Q*-test.

## Results

Twenty healthy volunteers randomized to treatment sequences completed three treatment conditions. The data obtained from cognitive testing were then analyzed.

**FIGURE 4:**
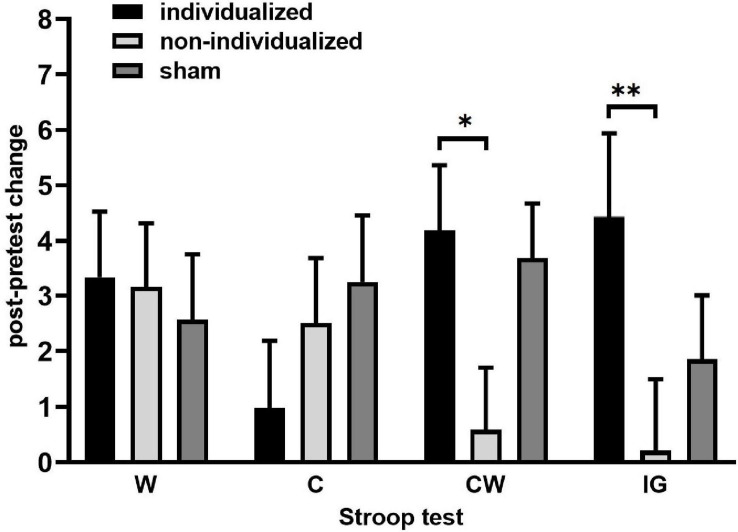
Post-modulation changes in the Stroop task after HD θ-tACS. Post-pretest change in the Stroop task represented by the black (W) and colored ink (C) congruous conditions, by the incongruous color-word condition (CW), and by the interference score (IG). The data are presented as mean ± SE. ^∗^*p* < 0.05 and ^∗∗^p<0.01 after Sidak’s correction, linear mixed-effects model.

The only significant treatment effect was found in the Stroop-CW subtest [*F*_(__2, 27.2__)_ = 4.47, *p* = 0.021] and Stroop-IG interference score [*F*_(__2, 28.7__)_ = 4.05, *p* = 0.028], whereas the effects of sequence [CW: *F*_(__5, 19.6)_ = 0.9, *p* = 0.5; IG: *F*_(__5, 16.4)_ = 0.6, *p* = 0.7], period [CW: *F*_(__2, 27.5)_ = 0.3, *p* = 0.7; IG: *F*_(__2, 27.7)_ = 1.5, *p* = 0.2], carryover [CW: *F*_(__2, 26.2)_ = 1.4, *p* = 0.3; IG: *F*_(__2, 27.2)_ = 0.7, *p* = 0.5], and treatment × period interaction [CW: *F*_(__4, 27.1)_ = 0.1, *p* = 1.00; IG: *F*_(__4, 27.9)_ = 0.4, *p* = 0.8] were non-significant. A significantly better performance was achieved after the individualized (rather than the non-individualized) tACS [CW: difference in least-square (LS) means = 3.89, 95%CI 0.30–7.46, *t* = 2.72, *p* = 0.03; IG: difference in LS means = 4.22, 95%CI 0.32–8.11, *t* = 2.71, *p* = 0.03], but when compared with the sham, neither the individualized nor the non-individualized tACS differed significantly (CW: individualized vs. sham: 0.47, 95%CI −3.19–4.12, *t* = 0.32, *p* = 0.9; non-individualized vs. sham: −3.40, 95%CI −0.16–6.98, *t* = −2.40, p = 0.07; IG: individualized vs. sham: 2.39, 95%CI −1.60–6.47, *t* = 1.18, *p* = 0.25; non-individualized vs. sham: −1.82, 95%CI −5.66d–2.02, *t* = −0.85, *p* = 0.4 (Table 2 and Figure 4).

**TABLE 2:** Post-modulatory effect of High Definition θ-tACS on cognitive performance.

**High definition θ-tACS**		**Individualized**		**Non-individualized**		**Sham**		Difference (*p*-value*)		
		**Pre-**	**Post-**	**Pre-**	**Post-**	**Pre-**	**Post-**	**I – Non-I**	**I – Sham**	**Non-I – Sham**
Visual Simon Task	Time k (ms)	402.7 (56.0)	392.5 (61.5)	416.9 (70.6)	397.9 (54.5)	393.9 (65.5)	389.7 (48.8)	0.93	0.94	0.65
	Time ink (ms)	443.6 (71.9)	423.9 (70.3)	430.2 (56.0)	415.9 (61.4)	424.6 (72.1)	418.3 (60.7)	0.94	0.66	0.92
Stop Signal Task	Time ns (ms)	603.1 (173.6)	578.9 (182.5)	602.5 (172.6)	579.3 (182.7)	550.8 (157.5)	567.3 (177.2)	0.99	0.82	0.66
	Time s (ms)	523.8 (139.5)	522.1 (173.8)	531.4 (161.9)	518.2 (160)	516.6 (162.8)	517.2 (163.9)	0.97	0.99	0.99
CPT III	Commissions	50.6 (9.9)	51.6 (10.3)	49.4 (9.9)	51.5 (11.5)	49.3 (9.4)	50.2 (10.0)	0.94	0.99	0.81
	Perseverations	46.3 (1.4)	47.7 (4.5)	46.6 (1.9)	47.5 (4.5)	46.2 (1.4)	48.4 (4.1)	0.86	0.59	0.32
	HRT (ms)	374.1 (31.6)	367.7 (28.0)	376.9 (32.7)	373.0 (33.9)	377.0 (29.8)	369.6 (29.0)	0.53	0.89	0.45
	HRT BC (ms)	1.5 (4.6)	1.9 (3.9)	0.01 (5.3)	2.1 (4.0)	−0.5 (4.5)	0.2 (3.7)	0.96	0.96	0.74
	Detectability	45.6 (6.8)	47.2 (8.4)	44.5 (6.9)	47.2 (8.8)	44.4 (7.1)	46.3 (8.0)	0.95	1.00	0.97
Stroop	C	84.8 (11.6)	86.2 (12.6)	83.8 (12.6)	86.2 (10.7)	83.1 (13.4)	86.0 (11.2)	0.52	0.41	0.36
	CW	55.6 (12.1)	59.7 (12.6)	587 (11.7)	59.4 (11.7)	59.1 (13.7)	62.8 (13.2)	0.02	0.50	0.13
	IG	8.5 (9.2)	13.0 (12.2)	12.2 (8.0)	12.3 (10.0)	13.1 (9.7)	14.9 (10.3)	0.01	0.26	0.39

The data are presented as a mean (and SD) of pre-test or post-test values respective θ-tACS protocols; * linear mixed-effects model, post-hoc pairwise comparisons with Sidak’s correction. I, Individualized θ-tACS montage of the mPFC-ACC. Non-I, Non-individualized (fixed) θ-tACS montage over the MFC. Visual Simon Task: Time k, mean reaction time for congruent stimuli; Time ink, mean reaction time for incongruent stimuli; Stop Signal Task: Time ns, mean reaction time for stimuli without noise; Time s, mean reaction time for stimuli with noise; CPT III, Continuous Performance Test III: Commissions = number of incorrect responses to non-targets “X”; Perseverations, number of perseverative responses; HRT, Hit Reaction Time; HRT BC, Hit Reaction Time Block Change; Detectability, ability to discriminate between targets “non-X” and non-targets “X”; Stroop Test: C, number of items properly named in 45 s in the color (C) congruous condition; CW, number of items properly named in 45 s in the color-word (CW) condition, where color-words are printed in an inconsistent color ink; IG, interference score. Data for Visual Simon Task and Stop Signal Task are presented as milliseconds (ms). Data for CPT III: Commissions, Perseverations and Detectability are presented as T-Scores; HRT and HRT BC are presented as milliseconds (ms).

Except for transient sensations of tingling or burning at the beginning of the tACS procedure, induction of phosphenes (visual sensations of a ring or spot) during the active protocols, and transient headache, HD θ-tACS was well-tolerated, with no serious adverse effects reported (Table 3). Interestingly, neuromodulatory conditions differed in emotional and mood effects at debriefing. Frequent irritation was reported after the non-individualized θ-tACS, while the individualized condition was associated more frequently with calm and harmonization (Table 3).

**TABLE 3:** Adverse or side effects, including bodily sensations and mood state changes induced by θ-tACS.

**Bodily sensations and mood states changes**		**Indi vidualized**	**Non-individualized**	**Sham**	*p*-value*
Adverse or side post-modulation effects	Mild Headache	1	2	0	0.22
	Irritability	1	9	0	<0.001
	Harmonization or Calm	8	1	0	0.001
	Increase of Energy	0	3	0	0.05
	Fatigue	1	0	0	0.37
Tingling or burning during tACS		15	15	14	0.37
Phosphenes during tACS		3	2	0	0.25

The data presents adverse or side effects, including bodily sensations and mood state changes in specific θ-tACS protocols during and after θ-tACS. The data show the number of participants who reported the symptoms. The cells “tingling” or “burning” refer to sensations on the skin in the area around the neuromodulatory electrodes at the beginning of θ-tACS application. *Cochrane Q-test.

When evaluating blinding integrity, the observed agreement was 50%, with kappa 0.24, SE 0.15, *z* = 1.58, *p* = 0.06. The participants did not estimate the order of treatments (a position of placebo) significantly better than by chance (34%), so a major violation of blinding was not detected.

## Discussion

The present study is the first to examine the influence of individualized HD θ-tACS targeted at the ACC on inhibitory control performance. We used the optimal electrode arrangement based on computational modeling from individual MRIs to target the current flow with the highest specificity at the mPFC-ACC, with maximum current density in the ACC, to investigate the post-modulatory θ-tACS effect on inhibitory control. Based on the evidence from previous studies that confirmed the alteration of inhibitory control performance during θ-tACS neuromodulation (van Driel et al., 2015; Moliadze et al., 2019), we extended the experiment to include a θ-tACS condition that modulated the ACC indirectly through MFC.

The post-modulatory effects of different θ-tACS protocols on inhibitory control performance were investigated with a novel paradigm of individualized HD θ-tACS, non-individualized θ-tACS targeting the ACC indirectly via the MFC, and a sham condition.

The main finding of our study was the presence of a post-modulatory effect in the improvement of inhibitory control with individualized HD θ-tACS. In contrast with the non-individualized θ-tACS, it demonstrated a post-modulatory improvement of inhibitory control with a verbal component, as documented within 45 min post neuromodulation. Specifically, we found a significant improvement in Stroop-CW and Stroop-IG performances following individualized HD θ-tACS. Unlike the parameters in other selected tasks, these tasks reflect the inhibitory control function with a verbal component. The changes in the non-verbal cognitive tasks (Simon Task, Stop Signal Task, and CPT III) did not induce a parallel effect. Compared with the sham condition, neither of the active θ-tACS protocols (individualized or non-individualized) were able to alter inhibitory control performance.

In general, our findings are in keeping with previous studies that have reported θ-tACS modulation of ACC activity (Chander et al., 2016; Onoda et al., 2017) and inhibitory control performance (van Driel et al., 2015; Moliadze et al., 2019).

Our results confirm those of a previous study in which the effect of alpha band (α) tACS on phonological word decisions was monitored (Moliadze et al., 2019). Although the authors used a different frequency (10 Hz rather than 6 Hz), bilateral prefrontal electrode positioning, and a different task for inhibitory control performance monitoring, their results showed a similar post-modulatory effect of tACS on inhibitory control with a verbal component. Interestingly, Fusco et al.’s (2020) study, in which they monitor the effect of tACS over the MFC and the extrastriate body area (with an electrode arrangement in the FCz and P08 areas) in cognitive conflicts related to bodily stimuli, highlights the causal relationship between function-specific brain areas and category-specific conflict tasks. During θ-tACS, there was an improvement in the Hand-Flanker task (representing bodily stimuli), but not in the Letter-Flanker task.

However, our results did not correspond with the findings of a study that used a non-individualized θ-tACS over the MFC (Fusco et al., 2018), though the authors only tested the immediate effect of a short-lasting (240 s duration) θ-tACS during the activation of the ACC with a specific inhibitory control task. This may interfere directly with cognitive processes and boost the effect of neuromodulation, similar to the effect shown in tDCS studies involving additional on-line cognitive training (Dedoncker et al., 2016; Hill et al., 2016). In contrast, and in keeping with van Driel et al. (2015), we used long-lasting θ-tACS to evaluate changes in the post-session processing of inhibitory control.

The finding that the non-individualized protocol was not effective in altering inhibitory control may be attributed to more complex regulation of inhibitory control than simple dACC activation. It also supports the assumption that the effect of θ-tACS may be affected by the concurrent modulation of surrounding and interconnected structures, by parallel modulation of the DLPFC in the individualized protocol, and by simultaneous modulation of the frontal and parietal cortex, the inferior frontal gyrus (IFG) in particular. This would explain why van Lehr et al.’s (2019) study, in which the DLPFC was modulated, and Moliadze et al.’s (2019) study, in which the prefrontal cortex was targeted bilaterally, showed a tACS effect in altering the verbal component of inhibitory control (though the results were inconsistent).

Interestingly, the present study did not show any sustained changes in non-verbal performance. We speculate that the absence of the effect on inhibitory control in non-verbal tasks after θ-tACS may be related to the greater stability of simpler non-verbal inhibition processes that are possibly more difficult to affect. Inhibitory control of verbal performance, where the ACC is also responsible for the semantic coding process in verbal working memory and is considered to be the central neural base of the central executive processes (Kaneda and Osaka, 2008; Reverberi et al., 2015), is a more complex function that may be more sensitive to the changes induced by neuromodulation, including the post-modulatory effect. An alternative explanation may be the use of different cognitive control strategies in the Stroop and Simon Task. Each of these tests bias attention toward a different approach in resolving the conflict. While the Stroop enhances the processing of task-relevant cues (i.e., it enhances the processing of colors and does not inhibit processing of irrelevant cues—namely, the words), the Simon Task inhibits task-irrelevant cues (i.e., it suppresses the position of the button, but does not enhance processing of the relevant cue—namely, the color of the square (Egner and Hirsch, 2005; Egner et al., 2007; Egner, 2008; Soutschek et al., 2013). Although both control strategies employ the same network (Peterson et al., 2002), the first modulates the activity in the parietal cortex, while the response-based conflict resolution modifies premotor cortex activity (Egner et al., 2007). This difference is consistent with Pratte et al. (2010), who found that the Stroop interference effect is smallest in fast responses and increases with longer response times, while the Simon’s effect is strongest in fast response time trials, and decreases as the response time increases. In the present study, the stimulation led to better performance in Stroop-CW (i.e., the Stroop effect was smaller), which meant a shorter RT per CW. This indicates a boost in the processing of task-relevant traits of the stimuli. On the other hand, no Simon effect modulation resulted in the improvement of response-selection processing. A possible explanation for this difference is the fact that partial (unintentional) stimulation of the premotor cortex may disrupt the overall effect in the Simon Task, but not in the Stroop.

A previous θ-tACS study that modulated PFC showed an altered processing in emotion evaluation/appraisal that corresponded to the integration function of the dACC (Onoda et al., 2017). Analogously, we observed unexpected mood changes induced by neuromodulation, where the volunteers reported irritation following the non-individualized protocol, and more often felt calm and harmonized after the individualized session (Table 3). We speculate that irritability after non-individualized θ-tACS may be mediated by the simultaneous modulation of the IFG and parietal region, which are not influenced by the individualized protocol. Therefore, non-individualized θ-tACS of the ACC may alter common brain activity (particularly motor and premotor) in other areas (Veniero et al., 2011; Alfonso et al., 2013), and this may impair the soothing effect of θ-tACS of the ACC. In contrast, a calm mood or mild fatigue induced by the individualized protocol may be attributed to simultaneous synchronization of the activity in the rostral ACC, which is associated with emotional processing (Bush et al., 2000).

For further exploration and verification of these observations, future protocols should include emotional tasks. For example, the emotional Stroop Test (Dresler et al., 2009), unlike the “standard” cognitive Stroop, is related to the rostral ACC activation, which was modulated simultaneously in both active protocols in the present study. It would also be beneficial to use specific scales to quantify emotional responses to neuromodulation.

To calculate individualized HD θ-tACS, we used high current density distribution to the mPFC-ACC along with low current density distribution in other cortical areas, including the parietal and temporal cortex. Electrode placement in the occipital area targeting the ACC via the mPFC also caused simultaneous neuromodulation of the visual cortex and may induce phosphenes, which could also be of a retinal origin (Kanai et al., 2010). Nevertheless, network analyses of inhibitory control-related network connections did not identify the occipital cortex as a region that is essential for inhibitory control (Spielberg et al., 2015). Moreover, the Stroop was measured before and after neuromodulation; therefore, visual performance during the cognitive tests could not be impaired.

In keeping with most previous studies testing the effect of θ-tACS on inhibitory control (van Driel et al., 2015; Lehr et al., 2019), our neuromodulation protocols used a total current of 1 mA. Fusco et al. (2018, 2020) administered a current of 1.5 and 2 mA, respectively. So far, the GTEN 100 used in our experiment has not allowed neuromodulation with a higher intensity of current. Previous findings have confirmed the dependence of tACS’s efficacy on current strength (Vosskuhl et al., 2016). It may be presumed that the upgrading of GTEN technology to higher current intensities will enhance the effect of neuromodulation.

Fusco et al. (2018) protocol used two circular sponge-conductive-rubber electrodes (Sponstim, 25 cm^2^, Neuroelectrics, Barcelona, Spain) placed over the MFC (in the FCz and Pz areas) for tACS. Our non-individualized protocol used MFC electrode layouts with 10 (five anode-cathodes; five cathode-anodes) circular electrodes (HCGSN 100, 1 cm^2^, EGI, Eugene, OR, United States), where each group of electrodes (in the FCz and Pz areas) covered 16–25 cm^2^, depending on the size of the subject’s head. The contact area of 5 cm^2^ was not changeable. The group of five electrodes was grouped in a square arrangement (four electrodes placed at the vertices of the square and the fifth central electrode in the FCz or Pz areas). Therefore, we cannot rule out the possibility that this methodological difference may lead to different results and outcomes.

The main shortcoming of the present study is the lack of neurophysiological evidence to confirm that the ACC was affected by tACS. Furthermore, individualized HD θ-tACS was applied via the mPFC, a cortical hub functionally connected with many associated brain areas, which might interfere with the effect. Therefore, we cannot rule out the possibility that the effect of individualized HD θ-tACS was influenced by concurrent modulation of the mPFC and its associated areas.

The study is limited by the lack of a control frequency condition. The testing could be enriched, in addition to the sham, by a control frequency condition to show that the detected behavioral changes are causally related to θ-tACS (6 Hz) and do not depend on other factors.

Future studies should also investigate the electrophysiological effects of θ-tACS (Bergmann et al., 2016; Cunillera et al., 2016; Neuling et al., 2017). In particular, it would be useful to monitor θ-tACS-induced changes in ERPs during performance tasks focused on cognitive functions. In the case of action monitoring, the suitable ERPs to study would be error-related negativity/error negativity (ERN; Gehring et al., 1995; Luu et al., 2000), or cognitive inhibition (N2; Yeung et al., 2004; Huster et al., 2013). Both, ERN and N2 are associated with the theta band (Luu et al., 2004; Cavanagh and Frank, 2014). In the latter, the nogo condition elicits a more negative response than the go condition (Lavric et al., 2004). Better performance in cognitive control tasks is associated with smaller N2 amplitudes (Lamm et al., 2006), while in ERN, the enhancement of negative amplitude is related to hyper-functioning error monitoring processes (Ruchsow et al., 2005).

## Conclusion

In conclusion, we have demonstrated that, compared with non-individualized θ-tACS, individualized HD θ-tACS applied via the mPFC-ACC significantly improves the post-tACS verbal component of conflict-processing. The proposed algorithm of the individualized HD θ-tACS confirmed better specificity in neuromodulation targeting. Our findings support the role of the ACC as the candidate region for inhibitory control processes and show that HD θ-tACS is a safe and well-tolerated method. Further studies examining the neuromodulation-induced changes in inhibitory control processes are warranted to verify the effect of the procedure and to optimize the neuromodulation parameters, including electrode assembly and current intensity. Future researchers should also investigate whether the individualized HD θ-tACS of the mPFC-ACC can modulate inhibitory control processes impaired by neuropsychiatric disorders associated with ACC disruption.

## Data Availability Statement

The raw data supporting the conclusions of this article will be made available by the authors, without undue reservation.

## Ethics Statement

The studies involving human participants were reviewed and approved by the Independent Ethics Committee of the National Institute of Mental Health, Klecany, Czechia. The patients/participants provided their written informed consent to participate in this study. Written informed consent was obtained from the individual(s) for the publication of any potentially identifiable images or data included in this article.

## Author Contributions

MK: conceptualization, methodology, investigation, project administration, visualization, writing – original draft preparation, and supervision. VV: conceptualization, methodology, investigation, project administration, and writing - reviewing and editing. JH: supervision and writing - reviewing and editing. PM: conceptualization and writing - reviewing and editing. JJ: investigation and writing – original draft preparation. DD: conceptualization, methodology, investigation, and writing - reviewing and editing. LK, SB, and OL: investigation and writing - reviewing and editing. DF: validation, data curation, and writing - reviewing and editing. TN: conceptualization, formal analysis, writing – original draft preparation, and writing - reviewing and editing. All authors contributed to the article and approved the submitted version.

## Conflict of Interest

The authors declare that the research was conducted in the absence of any commercial or financial relationships that could be construed as a potential conflict of interest.

## Footnotes

1 http://www.randomization.com
